# Booster vaccination: host preparation against Omicron challenge by innate immunity training

**DOI:** 10.1002/mco2.477

**Published:** 2024-01-30

**Authors:** Lan Bai, Fangfang Zhou, Long Zhang

**Affiliations:** ^1^ International Biomed‐X Research Center, Second Affiliated Hospital of Zhejiang University School of Medicine, Zhejiang University Hangzhou China; ^2^ Institutes of Biology and Medical Science, Soochow University Suzhou China

1

During the SARS‐CoV‐2 pandemic, multiple variants surmounted pre‐existing immune barriers, causing steady breakthrough infections in previously vaccinated individuals. Nevertheless, multiple‐dose vaccinations based on the SARS‐CoV‐2 prototype conferred protection during Omicron challenge.^1^ However, the cellular mechanisms remain unresolved. In a recent study published in *Cell*, Yu et al. revealed that booster vaccinations may prompt innate immunity training, benefiting host defence against Omicron challenge by facilitating the differentiation of HLA‐DR^hi^ classical monocyte (HLA‐DR^hi^‐CMs) and non‐classical monocytes (NCMs) from monocytes upon infection.^2^ Therefore, this study provided new insight into how booster vaccinations induce trained immunity against infection with SARS‐CoV‐2 variants.

Booster vaccinations can trigger strikingly long‐term immune and antibody responses before infection.^3^ To investigate how prior booster vaccinations based on the COVID‐19 prototype modulate human immune responses during Omicron challenge, Yu et al. enrolled 122 Omicron‐infected patients and 50 uninfected participants, who were either not vaccinated or pre‐vaccinated with different doses of inactive vaccines based on the COVID‐19 prototype. The participants were then divided into six groups, and a systemic analysis was performed on their circulating mononuclear cells and plasma cytokines using mass cytometry and Olink proteomics.

First, the authors investigated the in‐depth characteristics of both the lymphocytic and myeloid compartments across the six groups (cohort). In this regard, they found that levels of regulatory T cells (Tregs) and three subpopulations of CD4^+^‐naïve T cells were elevated by Omicron infection, whereas three‐dose booster vaccinations dampened these tendencies. Moreover, booster vaccinations increased the number of Th1 CD4^+^ T cells and Th1‐like effector memory CD4^+^ T cells. However, prior booster vaccinations did not change the frequencies of other lymphocytic subsets, such as CD8^+^ T, NKT, or B cells, during infection. Thus, prior booster vaccinations may benefit the expansion of CD4^+^ effector T cells from CD4^+^‐naïve T cells by curbing the increase in Tregs during Omicron infection.

Innate myeloid immune cells play a key role in the acute phase of viral infection. The authors also investigated the alterations in six myeloid clusters and observed that three subsets of effector monocytes decreased significantly upon Omicron challenge, suggesting that human innate immunity was suppressed by Omicron infection. Nevertheless, booster vaccinations significantly elevated the frequencies of NCMs and HLA‐DR^hi^‐CMs (Figure 1). Importantly, they found a positive correlation among HLA‐DR^hi^‐CM, pDC, and NCM subsets, whereas Tregs were negatively correlated with these subsets. Hence, prior booster vaccinations stimulate an innate immunity activation module.

**FIGURE 1:**
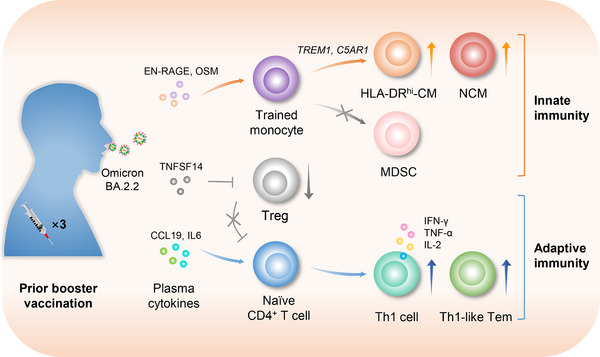
A prior three‐dose vaccination (booster vaccination) prepares hosts to defend against Omicron challenge by training both innate and adaptive immunity. Booster vaccinations induced a plasma microenvironment and a transcriptional signature of enhanced memory‐like innate response, facilitating the activation and differentiation of monocytes, rather than differentiation into MDSCs, during Omicron infection. Therefore, increased expansion of HLA‐DR^hi^‐CM and NCM effector monocytes and a milder symptom occurrence were shown in patients who had obtained booster vaccinations. Moreover, this trained immunity is positively correlated with naïve CD4^+^ T‐cell activation, but negatively correlated with aberrant Treg expansion. HLA‐DR^hi^‐CM: HLA‐DR^hi^ classical monocyte; NCM: non‐classical monocyte; MDSC: myeloid‐derived suppressive cell; Th1 cell: Th1 CD4^+^ T cell; Th1‐like Tem: Th1‐like effector memory CD4^+^ T cell; Treg: regulatory T cell.

Next, the authors comprehensively analyzed PBMCs by bulk mRNA‐seq to gain functional and mechanistic insight. To this end, the authors compared the vaccination‐induced significantly altered genes in uninfected participants with those in infected patients, and found a commonly regulated gene cluster that included *TREM1* and *C5AR1*, two key genes involved in the regulation of monocyte activation. In the vax3‐inf group, the expression levels of *TREM1* and *C5AR1* were significantly upregulated in the asymptomatic patients. Yu et al. also identified several top‐ranked molecular pathways by GSEA. Pathways associated with the activation of monocytes and effector memory T cells were activated by prior booster vaccinations during infection, whereas immunodeficiency and myeloid‐derived suppressive cell (MDSC) polarization activities were not upregulated by three‐dose vaccination, suggesting that specific trained pathways involved in monocytic activation that prepare hosts against Omicron infection could originate from prior booster vaccinations. Consistently, the percentage of asymptomatic patients was the highest in the three‐dose vaccination group. Moreover, it was shown that the frequencies of two subsets of effector monocytes and the percentage of asymptomatic patients were positively correlated with the key regulatory pathways that were significantly activated in the three‐dose vaccination group, whereas the activity of Tregs was negatively correlated with the key regulatory pathways upregulated by booster vaccinations. These findings suggest that prior three‐dose booster vaccination may prepare the host in coping with Omicron infection by promoting the activation and differentiation of trained monocytes into effector monocyte subsets (Figure 1).

Plasma is an important microenvironment influencing the immune state through abundant inflammatory factors. The authors systematically evaluated 180 cytokines in the plasma across the cohort, and identified several top‐ranked molecular pathways and cytokines associated with monocytic activation and Treg restraint. Accordingly, the expansion of two subsets of HLA‐DR^hi^‐CM and NCM effector monocytes was promoted in patients who had obtained prior booster vaccinations, suggesting that a plasma microenvironment that benefits monocyte activation and differentiation was induced by prior booster vaccinations (Figure 1).

In conclusion, this outstanding work by Yu et al. systematically investigated the human cellular and immune responses and mechanisms modulated by prior booster vaccinations, particularly innate immunity memory, during Omicron challenge. Briefly, booster vaccinations induced a plasma microenvironment and a transcriptional signature of enhanced memory‐like innate response, which was consistent with the enhanced activation and differentiation of monocytes during Omicron infection. Thus, patients who had received booster vaccinations showed increased expansion of HLA‐DR^hi^‐CMs and NCMs effector monocytes and milder symptoms (Figure 1).

Most vaccines require multiple doses to elicit long‐lasting immune memory protection, which has been classically attributed solely to adaptive immunity. However, recent studies have shown that human innate immunity is also equipped with adaptive characteristics after being challenged with pathogens, and can display long‐term protectional changes that increase nonspecific responses to subsequent infections. This phenomenon has been described as *innate immunity memory* or *trained immunity*.^4^

During the COVID‐19 pandemic, scientists reported a significantly enhanced innate immune response triggered by both booster BNT162b2 mRNA and SARS‐CoV‐2 inactivated vaccinations.^2^, ^5^ Moreover, the heterologous booster vaccinations demonstrated a better advantage in stimulating a faster and more robust humoral response than the homologous booster vaccinations did. However, both heterologous and homologous boosters can mount antibody responses persisting for over 6 months through metabolic reprogramming of B cells.^3^ Consistently, booster vaccinations and infection‐induced hybrid immunity showed strong protection against Omicron variants.^1^ Recently, Yu et al. found that booster vaccinations based on wild‐type SARS‐CoV‐2 can markedly induce innate immunity memory against Omicron challenge by enhancing the activation and differentiation of monocytes over the expansion of B, CD8^+^ T, and NKT cells.^2^ Whether a heterologous booster strategy might promote the activation and differentiation of monocytes during Omicron infection remains unclear, warranting further investigation into the durability and metabolic and epigenetic profiles of these trained monocytes. It is worth pointing out that monocytes in circulation and bone marrow undergo dynamic changes influenced by nutrition status; therefore, whether these alterations in immune cell frequencies are attributed to their migration behaviors needs to be ruled out.

The ongoing SARS‐CoV‐2 pandemic has raised more concerns about the effectiveness of current vaccination strategies. It is well known that the host defense system is shaped by the interaction of innate and adaptive immunity, and that the innate immune response is a prelude to the activation of B cells and T cells. Optimization of vaccination strategies requires a deeper knowledge of how different vaccination strategies affect innate immune cells, because trained innate immunity might be a game‐changer in booster vaccination strategies. Undoubtedly, this study by Yu et al. demonstrates that prior booster vaccinations based on prototype SARS‐CoV‐2 prime the innate immune system to mount a more potent response during Omicron challenge, providing valuable guidance for next‐generation booster strategies against unexpected viral outbreaks.

## AUTHOR CONTRIBUTIONS

Lan Bai and Long Zhang wrote the manuscript and prepared the figure. Fangfang Zhou provided valuable discussion. All authors have read and approved the final version of the manuscript.

## CONFLICT OF INTEREST STATEMENT

The authors declare they have no conflicts of interest.

## ETHICS STATEMENT

Not applicable.

## Data Availability

Not applicable.
